# Various Forms of Tissue Damage and Danger Signals Following Hematopoietic Stem-Cell Transplantation

**DOI:** 10.3389/fimmu.2015.00014

**Published:** 2015-01-28

**Authors:** Abdulraouf Ramadan, Sophie Paczesny

**Affiliations:** ^1^Department of Pediatrics, Melvin and Bren Simon Cancer Center, Indiana University, Indianapolis, IN, USA; ^2^Department of Microbiology and Immunology, Indiana University, Indianapolis, IN, USA

**Keywords:** graft-versus-host disease, danger signals, tissue damage, alarmins, pathogen-associated molecular patterns, damage-associated molecular patterns, innate immunity, biomarkers

## Abstract

Hematopoietic stem-cell transplantation (HSCT) is the most potent curative therapy for many malignant and non-malignant disorders. Unfortunately, a major complication of HSCT is graft-versus-host disease (GVHD), which is mediated by tissue damage resulting from the conditioning regimens before the transplantation and the alloreaction of dual immune components (activated donor T-cells and recipient’s antigen-presenting cells). This tissue damage leads to the release of alarmins and the triggering of pathogen-recognition receptors that activate the innate immune system and subsequently the adaptive immune system. Alarmins, which are of endogenous origin, together with the exogenous pathogen-associated molecular patterns (PAMPs) elicit similar responses of danger signals and represent the group of damage-associated molecular patterns (DAMPs). Effector cells of innate and adaptive immunity that are activated by PAMPs or alarmins can secrete other alarmins and amplify the immune responses. These complex interactions and loops between alarmins and PAMPs are particularly potent at inducing and then aggravating the GVHD reaction. In this review, we highlight the role of these tissue damaging molecules and their signaling pathways. Interestingly, some DAMPs and PAMPs are organ specific and GVHD-induced and have been shown to be interesting biomarkers. Some of these molecules may represent potential targets for novel therapeutic approaches.

## Introduction

Hematopoietic cells that are capable of self-renewing and reconstituting all types of blood cells along with allogeneic donor T-cells can be used to treat numerous malignant and non-malignant lethal diseases, including leukemias, lymphomas, inherited genetic diseases, and immune deficiencies. However, the success of allogeneic hematopoietic stem-cell transplantation (HSCT) is unfortunately limited by transplant-associated toxicities related to the applied conditioning regimens and the immunologic consequence of donor T-cell recognition of recipient alloantigens, which causes graft-versus-host disease (GVHD). Acute GVHD is characterized by selective tissue damage to the mucosa, particularly of the skin, gastrointestinal (GI) tract, and liver. Other tissues and organs such as the bone marrow, thymus, lungs (1, 2), and brain (3) have also been shown to be potential GVHD targets. Chronic GVHD not only targets organs, including the ones mentioned above, but also can damage the connective tissue and exocrine glands.

**Figure 1:**
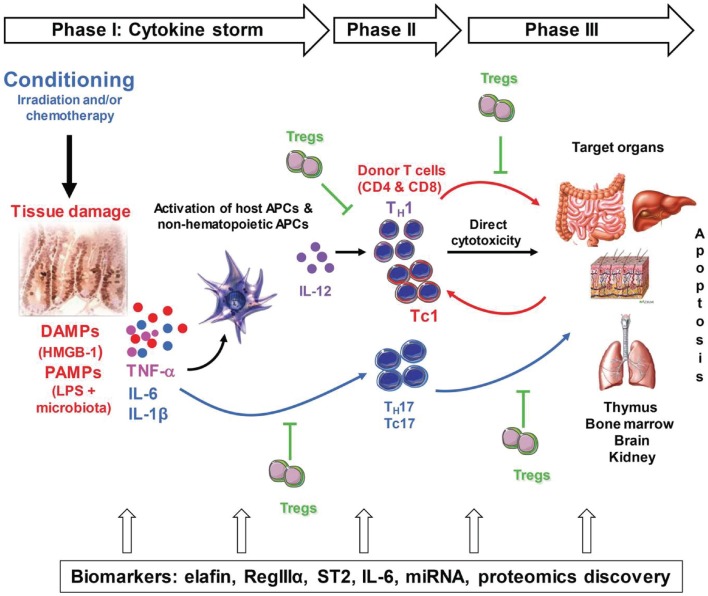
**Pathogenesis of acute GVHD**. Conditioning by irradiation and/or chemotherapy causes tissue damage. Damaged tissues and cells release DAMPs (HMGB-1), PAMPs (LPS) from gut microbiota as well as inflammatory cytokines such as IL-1β, IL-6, and TNF-α, which contribute to the “cytokine storm.” These are the first danger signals that activate host APCs, which activate and polarized donor T-cells toward pathogenic T-cells (TH1 and TH17 for CD4 and TC1, TC17 for CD8). Activated pathogenic T-cells infiltrate target organs (i.e., GI tract, liver, skin) and amplify local tissue destruction. The presence of regulatory T-cells (Tregs) helps reduce GVHD severity through the inhibition of pathogenic cells activation and/or expansion at early or further phases of GVHD. Some of these DAMPs and PAMPs such as elafin (skin-specific), regenerating islet-derived 3-alpha (REG3α, gut-specific), and suppressor of tumorigenicity 2 (ST2, a member of the IL-1 receptor family, binding IL-33) have been shown to be biomarkers.

The pathogenesis of GVHD can be summarized in three sequential steps: first, the conditioning regimen damages the tissues, causing production of danger signals, which are detailed in this review, and pro-inflammatory cytokines such as tumor necrosis factor (TNF)-α, interleukin (IL)-1, and IL-6. The culmination of these events is what the field refers to as the “cytokine storm,” which activates host antigen-presenting cells (APCs) and the newly infused donor T-cells. The second phase involves proliferation and differentiation of donor T-cells in response to host APCs, which results in rapid intracellular biochemical cascades that induce production of T helper (TH) 1, TH17 (for CD4 T-cells), T cytotoxic (TC) 1, and TC17 cells (for CD8 T-cells) that secrete cytokines such as interferon (IFN)-γ, IL-2, IL-17, and TNF-α. The last step is a complex cascade of cellular mediators and soluble inflammatory molecules that work synergistically to amplify local tissue injury. These mediators further amplify inflammation and target tissue destruction. GVHD is also characterized by an imbalance between the effector T-cells and the regulatory T-cells (Tregs). At all of these steps, the inflammatory cascade and various types of tissue damage lead to the release of biomarkers of GVHD into the blood, the detection of which can be achieved via blood tests. Markers such as elafin (skin-specific), regenerating islet-derived 3-alpha (REG3α, gut-specific), suppressor of tumorigenicity 2 (ST2, a member of the IL-1 receptor family, binding IL-33), and others are detailed in this review. Figure 1 summarizes these events.

## Danger Signal Proteins Following HSCT

Following conditioning (radiation and/or chemotherapy), exogenous and endogenous “danger” signals released from damaged tissues orchestrate mesenchymal, epithelial, and immune cellular communications to attempt to restore homeostasis. These danger’ signals induce rapid changes in redox-sensitive proteins, leading to the activation of nuclear transcription factors including nuclear factor (NF)-κb (4), early growth response factor (Egr1), and activator protein (AP)-1 (5), which are heavily involved in inflammatory cytokine production. Indeed, both radiation/chemotherapy effects and pro-inflammatory cytokines generate free reactive oxygen species (ROS) and reactive nitrogen species (RNS) such as superoxide, nitric oxide, hydroxyl radicals, peroxynitrite, and their products (6). Furthermore, inflammatory cytokines, including those of the IL-1 family, and TNF-α (7) require ROS for their activation. In contrast, anti-inflammatory cytokines [transforming growth factor (TGF)-β, IL-10, and IL-4] inhibit ROS/RNS-mediated effects and display anti-oxidative properties (8–10). Therefore, inflammatory and anti-inflammatory cytokines mutually influence each other through the production of ROS/RNS. Recently, it has been shown that mice exhibiting overexpression of ROS (mice deficient for negative regulator of ROS) develop more severe disease in an experimental autoimmune encephalomyelitis model (11), whereas mice deficient in ROS (NOX2 knockout mice) show less infiltration of neutrophils into the ileum and less tissue damage, leading to less severe GVHD (12).

Damage-associated molecular patterns (DAMPs) include exogenous pathogen-associated molecular patterns (PAMPs) as well as endogenous alarmins, each of which play a crucial role in the initiation of GVHD and are described in detail in subsequent paragraphs.

### Exogenous PAMPs during inflammation and GVHD

Early studies in allogeneic murine chimeras induced by radiation showed that the mortality due to “secondary disease,” later called GVHD, was significantly reduced in germ-free mice compared to conventional mice (13). Treating conventional mice with antibiotic prophylaxis also significantly delay mortality in comparison to that in the control group (14). Clinical studies have demonstrated the efficiency of GI decontamination in reducing GVHD (15, 16). PAMPs are conserved microbial molecules released by invading microorganisms (17, 18). They recognize pattern recognition receptors (PRRs), primarily toll-like receptors (TLRs), members of the cytosolic retinoic acid-inducible gene-I-like helicase family (19), and receptors with a nucleotide-binding domain (NOD) and leucine-rich repeats (NOD-like receptors, NLRs). These activate different pathways, resulting in the production of inflammatory cytokines through NF-κb activation. The main link between the PRRs and NF-κb activation/cytokine production during GVHD is the cytoplasmic myeloid differentiation primary response protein 88 (MyD88) in APCs (20). APCs, particularly recipient dendritic cells (DCs), primed by the conditioning are potent sensors of PAMPS, which leads to their activation and augmented major histocompatibility complex (MHC) presentation to T-cells (21). Similar to TLRs, the NLR family has an impact on GVHD. The absence of NOD2 in recipients results in more severe GVHD in both MHC-mismatched and MHC-matched models (22). Single nucleotide polymorphisms of NOD2/CARD15 have also been associated with severe GVHD in patients receiving stem cells from either human leukocyte antigen (HLA)-identical or unrelated donors (23).

### Alarmins and endogenous DAMPs during inflammation and GVHD

The term “alarmins” is used to describe the endogenous molecules equivalent to PAMPs. They rapidly produce a danger signal after non-programed cell death or a specific modality of programed cell death (24). More recently, they are increasingly referred to as DAMPs, in reference to the term PAMPs, because they share structural and functional similarities with exogenous PAMPS. However, this definition of DAMPs is not used consistently, and sometimes endogenous alarmins and exogenous PAMPs are classified together as DAMPs.

#### High mobility group box 1

High mobility group box 1 (HMGB-1) is a nuclear protein that binds to nucleosomes and promotes DNA bending (25). It is expressed in most cells but at varying levels, and it is also present in the extracellular milieu after non-programed cell death (26, 27). It can be released in tumors (28). When under oxidative stress induced by irradiation, HMGB-1 acts as a DAMP and can mediate endotoxin lethality in mice (29). Persistence of a high level of HMGB-1 has been reported in chronic inflammatory disorders such as autoimmune disease (30), ischemia, and reperfusion injury (31). Targeted knockout or inhibition of HMGB-1 was shown to be able to increase apoptosis and suppress pancreatic cancer cell growth (32, 33). HMGB-1 has chemotactic activity toward monocytes, macrophage, neutrophils, and DCs (34, 35) in response to inflammatory cytokines such as TNF-α, IL-1, and IFN-γ (29, 36) as well as via reaction with TLR2/4 (37). It has been reported that certain donor HMGB-1 polymorphisms are associated with increased chronic GVHD, whereas recipient with the 2351 insT showed reduced risk of grade II–IV acute GVHD (38). A recent study showed that irradiation downregulates the HMGB-1 receptor (Siglec-G), which makes HSCT recipients more susceptible to GVHD. Moreover, addition of CD24 (Siglec-G ligand) alleviated GVHD in both minor and major-mismatched murine models of allogeneic bone marrow transplantation (39).

#### S100 proteins

S100 proteins are among more than 20 members of a family of low molecular weight proteins (9–13 kDa). They are produced as monomers and form dimers or multimers spontaneously (40, 41) following calcium binding protein activation (42). The most studied members in this family are S100A7, S100A8, S100A9, S100A12, and S100A15, which are mainly expressed in phagocytes, where they show high antimicrobial activity (43). They are thus released in inflammatory sites (44). Both S100A7 and S100A15 are induced by TH1, TH17, and TH22 cytokines and play an important role in psoriasis pathogenicity and act as alarmins in priming keratinocytes, thereby enhancing IL-6, IL-8, and TNF-α production and amplifying inflammation in the skin (45). On the other hand, S100A8 and S100A9 show a pathogenic role in lung inflammation mediated by neutrophil recruitment (46). Another member, S100A12, has been shown to be positively correlated with increased *Escherichia coli* colonies in infants via disturbance of the homeostasis between the intestinal microbiome and host immunity (47). No studies have shown the impact of this family in GVHD yet, but all of the above findings suggest that these DAMPs may play a role in different types of tissue damage and the pathology of skin and GI GVHD. Moreover, proteomic analysis of saliva showed that healthy controls have low or non-detectable levels of S100A9 and S100A8 proteins, whereas patients after HSCT without GVHD showed augmented levels of these proteins. Interestingly, patients with GVHD show higher levels of S100A8 and S100A9 than patients without GVHD (48). Moreover, a new study found that released S100 proteins are involved in the pathogenesis of GI GVHD through stimulation of monocytes, which enhance TH17 cells in patients receiving allogeneic HSCT (49).

#### Elastase inhibitors (endogenous proteases inhibitors)

During infection, the activity of locally produced mucosal alarm antiproteases, such as elafin and secretory leukocyte peptidase inhibitor (SLPI), may add an extra edge to the host defense (50). SLPI and elafin alarm antiproteases have been isolated and characterized under a variety of names in adult and fetal tissues (51). They belong to the family of whey acidic proteins (WAPs). Elafin was isolated from the skin of psoriasis patients (52) and is produced by both epithelial cells and immune cells. Alarm antiproteases are generated locally in areas of infection or neutrophil infiltration and are upregulated by pathogen- and inflammation-associated factors, including cytokines and neutrophil elastases (NEs) (53). Elafin and SLPI have been proposed to possess “defensin/cathelicidin-like” properties (54). It has been shown that in the 117 amino acids encoded by the elafin gene, the first 22 amino acids represent hydrophobic signal peptide. Elafin is produced as a 9.9-kDa full-length non-glycosylated cationic protein (55, 56). Elafin expression *in vitro* can be enhanced by adding inflammatory cytokines (IL-1 and TNF-α) to cultured bronchial and alveolar epithelial cells (57). These cytokines induce a similar increase in elafin expression by keratinocytes *in vitro* (58). Interestingly, these cytokines increase expression of elafin more than that of SLPI *in vitro* (57). Thus, elafin may have greater significance during an inflammatory challenge to the lung, in keeping of the notion that elafin mRNA expression in bronchial epithelial cells is increased by free NE, which is found in abundance during inflammatory events (53, 59). In addition to its NE inhibitory and immunomodulatory activities, elafin possesses broad-spectrum antibacterial, antiviral, and antifungal properties. Elafin expression is increased in the plasma of patients with skin GVHD compared to that of patients without GVHD following allogeneic HSCT without T-cell depletion. Moreover, elafin concentrations have been positively correlated with the grade of skin GVHD. Importantly, elafin is not elevated in rashes caused by conditions other than GVHD, making it a specific biomarker for skin GVHD (60). This is because elafin is induced by inflammatory cytokines, which mediate GVHD by targeting keratinocytes (61).

#### Defensins

The defensins are short peptides with a characteristic β-sheet-rich fold and, like SLPI and elafin, are cysteine-rich, containing multiple disulfide bonds (62, 63). Defensins are classified into three subfamilies (α, β, θ). The α-defensins are neutrophil peptides [human neutrophil peptides (HNPs) 1–4]. In humans, α-defensins [human defensin (HD)-5 and HD-6] are mostly expressed in Paneth cells in the small intestine (64, 65). HNPs 1–3 are expressed in B-cells, γδ T-cells, natural killer (NK) cells, and DCs. α-defensins exhibit wide spectrum antimicrobial coverage against Gram negative and Gram positive organisms and also have some antifungal activity against *Candida albicans*, as one example. β-defensins (four diverse human β-defensins, HBD 1–4) have been classified based on their function and genomic targeting. They are expressed by epithelial cells, macrophages, macrophage derived DCs, and monocytes (66). In murine models of haploidentical or minor mismatched BMT, it has been shown that the reduction in α-defensins following GVHD damages Paneth cells, resulting in a loss of variation in the gut microbiota composition (67). Consistently, clinical data show significant increases in *Lactobacillales* and decreases in *Clostridiales* in patients with GVHD after allogeneic HSCT (68, 69). In this cohort, overall survival was significantly worse in patients with lower intestinal diversity at engraftment as compared to intermediate and high diversity groups, respectively, even after adjustment for other clinical predictors (69).

#### Cathelicidins

Cathelicidins, which are recognized as a constitutive component of myeloid-derived cells (70), are the second major family of antimicrobial peptides (AMPs). Cathelicidins are highly heterogeneous (71–73), and as mediators of an effective system of host defense, they provide protection to intestinal epithelial cells against invading microorganisms and control the overgrowth of commensal bacteria (54). The human cathelicidin hCAP-18/LL-37 has a C-terminal peptide of α-helical type, which is present in all mammals. The widespread presence of this C-terminus suggests that the α-helical cathelicidin type is the progenitor molecule of this family (74). Even though it is considered a neutrophil-specific constituent distributed in all tissues, which is liberated as LL-37, it has also been shown to be produced by different immune cells, such as NK cells, γδ T-cells, B-cells, monocytes, mast cells, and immature neutrophils (75–77). LL-37 plays an important role in the prevention of oral bacterial infection, and it was found to be down-regulated in gut biopsies of patients infected with *Shigella* (78, 79). LL-37 expression is also induced after skin injury (80). In addition, its overexpression in human bronchial xenografts preserves the cystic fibrosis-specific bacterial killing defect (81). Recently, LL-37 has been shown to protect against arthritis in murine models through IL-32 suppression, and this observation was confirmed in human peripheral blood mononuclear cells (PBMCs) through decreases in pro-inflammatory cytokines such as TNF-α and IL-1β (82). In contrast, it also mediates immune cell recruitment by promoting chemotaxis, autophagy, and phagocytosis (83–87). It also enhances the adaptive immune responses (88, 89). LL-37 expression is decreased in Crohn’s disease and dermatitis, but elevated in psoriasis and systemic lupus erythematosus (90–92).

#### The regenerating protein family

The regenerating (Reg) III proteins were discovered in 1984 in pancreatitis experimental models (93). Later studies showed the presence of homologous proteins in human and mice (94–96). There are three different types of Reg III genes in mice (97) and all type III Reg genes appear to have diverged from a common ancestral gene. RegIIIα, RegIIIβ, and RegIIIγ have 60–70% homology and are all expressed in the intestine (97, 98). Reg1α expression is increased in inflamed colonic mucosa and correlates with IL-22 expression (99). In inflammatory bowel disease (100), it has been reported that Reg3α in humans or RegIIIγ, the homologous mouse protein, has an antimicrobial function, controlling bacterial proliferation (101, 102). In addition, following skin injury, RegIIIα expression increases in keratinocytes in response to IL-17 (103). In a haploidentical murine model of GVHD, it has been shown that RegIIIγ is upregulated, and this upregulation is not due to radiation-induced damage but due to the allogeneic response (104). Reg3α concentrations in plasma are increased by threefold in patients with GI GVHD compared to all other patients, including patients with non-GVHD enteritis following allogeneic HSCT. Reg3α expression is also positively correlated with GI GVHD grade and volume of diarrhea, suggesting that Reg3α represents a biomarker for the diagnosis of GI GVHD (105).

#### Heat shock proteins

The heat shock proteins (HSP) are a family of proteins that have an essential role as molecular chaperones, facilitating protein folding and intracellular transport (106). Expression of these proteins increases under various stress conditions such as infection, hypoxia, trauma, or exposure to toxic drugs, and high levels of HSPs are released by necrotic cells (107, 108). HSP60 (60 kDa) is expressed mainly in mitochondria and on the cell surface of monocytes after IFN-γ stimulation as well as on apoptotic T-cells (109). HSP60 also is overexpressed in intestinal epithelium in Behcet’s disease and in keratinocytes in skin lesions (110, 111). CD14/TLR acts as a coreceptor for HSPs (112). HSP70 expression correlates positively with GVHD grade (113). Another study showed that antibodies to 70 and 90-kDa HSPs are associated with GVHD in patients receiving allogeneic peripheral blood stem-cell (PBSC) transplantation (114).

#### Heparan sulfate proteoglycans

Heparan sulfate proteoglycans are proteins carrying one or more covalently bound heparan sulfate chain, a large anionic polysaccharide of glycosaminoglycan. These proteins show considerable diversity and interactive properties and are widely found in tissues within the extracellular matrix and are also found intracellularly (115). Functionally, heparin sulfate proteoglycans play critical roles in (i) mediating the formation of chemokine gradients for cell migration (116, 117); (ii) protecting cytokines such as IFN-γ against proteolysis (118); (iii) controlling the diffusion of their ligands (119). Heparan sulfate has also been shown to act as an endogenous TLR4 ligand and is a potent stimulator of T-cell alloreactivity *in vitro* (120). This action is dependent of the TLR4 pathway in DCs, but not in T-cells. Serum levels of heparan sulfate are elevated at the onset of GVHD and correlate to disease severity in an allogeneic mouse model and in patients received allogeneic HLA-matched HSCT (121).

#### Adenosine triphosphate

Adenosine triphosphate (ATP) is an essential purine base required for almost all physical responses. Extracellular ATP released from injured but not apoptotic cells is secreted rapidly after irradiation and mediates cellular responses through activation of purinergic receptors, which activate calcium channels (122). ATP activates caspase-1 and produces IL-1β, depending on the NLRP3 inflammasome (123). In GVHD, ATP is an endogenous danger signal released from necrotic cells (124, 125). Accumulation of ATP leads to upregulation of co-stimulatory molecules (e.g., CD80, CD86) *in vitro* and *in vivo*, which activates pathogenic donor T-cells and reduces the number of Tregs, resulting in greater production of inflammatory cytokines and aggravation of GVHD severity in an allogeneic murine model (126).

#### Uric acid

Uric acid is a metabolite of purine nucleotides in humans and has been described as a DAMP released from dying cells (24). Soluble uric acid induces the production of inflammatory cytokines such as monocyte chemotactic protein-1 in rat vascular smooth muscle cells (127) and is recognized by TLR2 and TLR4 and signaled through MyD88 (128). It can also be sensed by NLRP3, a member of the NLRs, and induces IL-1β production via caspase-1 activation (129). Injection of uric acid into mice along with antigen results in activation of CD8 T-cells, whereas abolition of uric acid inhibits the cytotoxicity of T-cells (130). *In vitro* addition of uric acid upregulates co-stimulatory molecules on bone marrow-derived DCs and leads to T-cell activation (131). In patients with acute GVHD showing high levels of uric acid in serum during the pre-transplantation period, inhibition of uric acid activity may be one aspect of the treatment strategy for reducing the severity of GVHD as is discussed later in this review (132).

## Danger Signaling Pathways in GVHD

**Figure 2:**
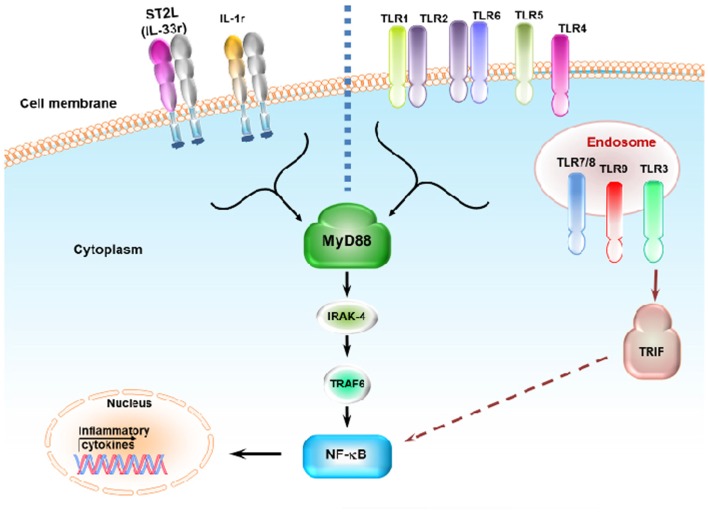
**Toll-like receptors and IL-1 receptor family signaling pathways**. ST2L (IL-33r) and IL-1r signal through the MyD88, IRAK4, and TRAF6 pathway. ST2L and IL-1r share this pathway with most TLRs. Binding of ST2L, IL-1r, and TLRs activates NF-κb, resulting in the release of inflammatory cytokines. Most TLRs signal through MyD88 expect for TLR3, which signals through the TRIF pathway. TLR4 can signal through both MyD88 and TRIF. TLR3, TLR7/TLR8, and TLR9 are expressed in the endosome while other TLRs are expressed on the cell surface. TLR1 and TLR6 recognize their ligand with TLR2 heterodimers.

**Figure 3:**
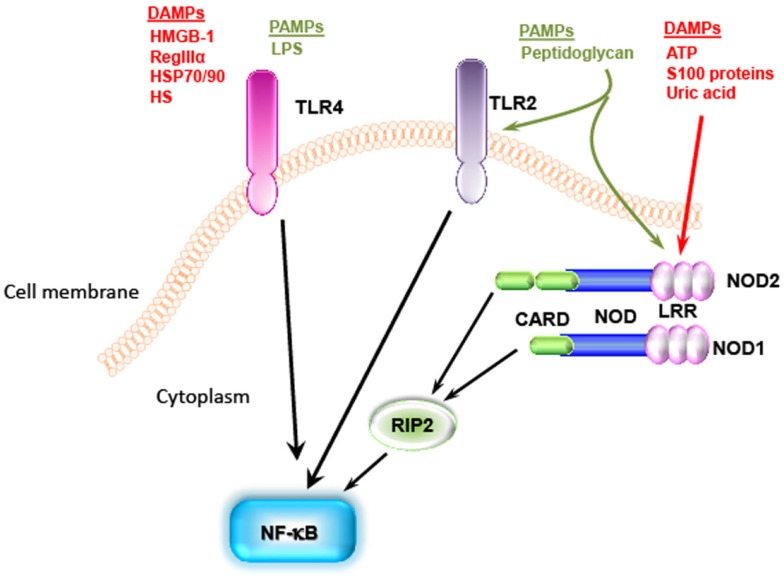
**Pathogen-associated molecular patterns and DAMPs share the same pathogen recognition receptors**. TLR4 recognizes LPS from Gram negative bacteria as well as DAMPS [i.e., HMGB-1, heat shock proteins (HSP)70/90, and heparan sulfate]. NOD can recognize peptidoglycan, which is known as TLR2 ligand, and some DAMPs such as ATP, S100 proteins, and uric acid. These signaling pathways all end with activation of NF-κb.

Individual danger signaling pathways involved in GVHD are detailed below and summarized in Table 1. Figure 2 summarizes TLRs and IL-1 receptor family signaling pathways. Figure 3 summarizes PAMPs and DAMPs common pathogen recognition receptors and their interactions with the signaling pathways.

**Table 1:** **GVHD-related PAMPs and DAMPs along with their signaling pathways and effects in GVHD**.

		Signaling pathway	Effect	Reference
PAMPs	Lipopolysaccharide	TLR4/MyD88 or TRIF	Aggravation	(133)
	TLR7 ligand (3M-011)	TLR7/MyD88	Aggravation	(134)
	Flagellin	TLR5/MyD88	Reduction (mouse)	(135)
	Intestinal microflora	TLR/MyD88	Aggravation (translocation)	(136, 137)
	Peptidoglycan	TLR2/MyD88 or NOD1	Not yet studied	(138, 139)
DAMPs	ATP	NOD2	Aggravation	(126)
	S100 proteins	NOD2	Aggravation	(48, 49)
	HMGB-1	TLR2/4/MyD88	Aggravation	(37)
	Reg III proteins	IL-22/IL-17/IL-1 family	Marker of intestinal GVHD	(104, 105)
	HSP	CD14/TLR4/MyD88	Aggravation	(112)
	Heparan sulfate	TLR4/MyD88	Aggravation	(120)
	Uric acid	NOD2/NLRP3	Aggravation	(129)
	Elafin	NF-κB	Marker of skin GVHD	(60)
	Defensins	Secreted	Protection	(67)
	sST2 (IL-33r)	MyD88	Marker of treatment refractory GVHD	(140)

### TLR signaling

The TLR family proteins are transmembrane receptors that were first described in humans in 1994 (141). In 1997, TLR4, which senses lipopolysaccharide (LPS) and activates innate cells, was described (142). TLRs play a key role in innate immunity by recognizing PAMPs. TLRs also recognize endogenous DAMPs (143). This family consists of 10 functional members in humans, where they are expressed on hematopoietic and non-hematopoietic cells (144). Their expression is either on the cell surface (TLR1, TLR2, TLR4, TLR5, and TLR6) or in endosomes (TLR3, TLR7, TLR8, and TLR9). Different TLRs recognize different PAMPs or DAMPS specifically. Among the surface TLRs, TLR2 recognizes Gram positive lipoprotein [such as peptidoglycan (PGN)], and TLR4 with its coreceptor MD-2 recognizes the Gram negative component of the cell wall LPS. TLR5 recognizes bacterial flagellin, and TLR1 and TLR6 form a dimer with TLR2 to recognize PGN. The other members of the family are expressed in endosomes and recognize viral or bacterial nucleic acid components: TLR3 recognizes double-stranded viral RNA; TLR7 and TLR8 recognize single-stranded viral RNA; and TLR9 recognizes bacterial or viral RNA and CpG-containing DNA (145–152). TLR4 also recognizes DAMPs such as the HSP family (HSP70 and HSP90) and heparan sulfate proteoglycans. Upon activation, TLRs transmit the signal through adaptor molecules shared with the IL-1R family myeloid differentiation factor 88 (MyD88) with the exception of TLR3, which signals through toll-IL-1 receptor (TIR) domain-containing adapter inducing IFN-β (TRIF). Only TLR4 can signal through both MyD88 and TRIF (143). Stimulation of TLRs activates NF-κB through a signaling cascade that is mostly MyD88-dependent. TIR adaptor protein (TIRAP) and IL-1 receptor-associated kinase 4 (IRAK4), which interact with TNF receptor-associated protein 6 (TRAF6), result in activation of mitogen-activated protein kinase (MAPK) and NF-κB signaling, leading to inflammatory cytokine production (153). On the other hand, TLR3 signaling, which is independent of MyD88 but dependent on TRIF, requires translocating chain-associating membrane protein (TRAM) for this signaling and activates TRAF3 (154). Stimulation of innate cells (e.g., DCs, macrophage, monocytes, neutrophils, and mast cells) via TLRs results in maturation, upregulation of MHC expression as well as that of co-stimulatory molecules (CD80/CD86, and CD40), and production of pro-inflammatory cytokines such as IL-12, TNF-α, and IL-6 (138, 155–157). This results in T-cell activation and proliferation. The inflammatory environment generated by TLR responses polarizes the response toward TH1, suggesting that the absence of TLR signaling may reduce GVHD severity. This was shown in a murine experimental model; mice deficient for TLR4 showed less severe GVHD than wild-type mice (133). Moreover, clinical studies showed association of TLR4 polymorphisms with GVHD severity (158). Other clinical studies of patients who received Tregs with their allo-HSCT showed significant augmentation of TLR5 expression, which correlated with GVHD severity (159). In contrast, an allogeneic BMT murine model demonstrated that treating mice with flagellin improves overall survival and suppresses GVHD (135). Likewise, TLR7 signaling was shown to increase donor T-cell infiltration when using R-848, a TLR7 ligand. The timing of administration of TLR7 ligands is important for GVHD pathogenesis as when using another TLR7 ligand (3M-011), as repetitive administration aggravates GVHD in an allogeneic mouse model (134). TLR9 signaling was shown to be important for GVHD pathogenicity as TLR9-deficient mice developed less severe GVHD and experienced increased survival. This effect was achieved through the production of less IFN-γ by host APCs (136, 160). Moreover, repeated treatment with CpG (a TLR9 ligand) increased mortality and GVHD severity (134). One clinical study showed no differences in the incidence and severity of GVHD between patients with gene variants associated with TLR9 reduction and controls (161), but another study reported severe acute GVHD when patients received stem cells from an unrelated donor with the A1174 gene. The T1635C variant was found to have a protective effect against GVHD (162). Thus, the roles of TLRs in GVHD remain controversial, according to differences in the timing of administration, experimental settings, microbiota constitution, and other alternative danger signaling pathways.

### NLR signaling

Another family associated with PRRs is the cytoplasmic NLR family, which was first described in 1999 and 2001 studies of NOD1 (CARD 4) (163) and NOD2 (CARD 15) (164), respectively. NOD1 was shown to bind to d-gamma-glutamyl diaminopimelic acid derived from peptidoglycan (139), and NOD2 binds to muramyl dipeptide (MDP) (165). Activation of NOD1 and NOD2 enables the recruitment of kinase receptor-interacting protein 2 (RIP2) (RICK) through caspase recruitment domain (CARD)–CARD homotypic interaction (166). RIP2 engagement by NOD receptors leads to ubiquitination of K63-linked by cellular inhibitors of apoptosis (cIAP)1 and cIAP2 (167), followed by recruitment of the TAK1/TAB2/TAB3 kinase complex to RIP2. X-linked inhibitor of apoptosis protein (XIAP) interacts with RIP2 and results in recruitment of the platform for the linear ubiquitination assembly complex (LUBAC), which mediates NF-κB activation. This signaling pathway converges on the induction of pro-inflammatory cytokines and initiates the innate immune response (168). Mutation in NOD2 leads to an inability for NF-κB activation after MDP stimulation in intestinal epithelial cells (169). However, NOD1 and NOD2 are able to induce direct autophagy through interaction with ATGL16L1, and this mechanism is independent of both RIP2 and NF-κB (170). NOD2 showed the capacity to recognize single-stranded RNA virus and to elicit interferon regulatory factor 3 activation, producing IFN-β through interaction with anti-viral signaling factor mitochondrial adaptor proteins (171). NOD2 also plays an important anti-viral role via CD8 T-cell priming in influenza A virus infection (172). Other studies of the expression of NOD2 on CD4 T-cells showed that the absence of NOD2 signaling impairs TH1 proliferation and response upon *Toxoplasma gondii* infection (173). Interestingly, the NLR family not only recognizes PAMPs but also recognizes DAMPs, such as ATP and DNA released from dying cells and uric acid (174, 175). Activation of the NLR family occurs via the inflammasome, cleavage of caspase-1, and production of active IL-1β and IL-18 (176). In GVHD models, the absence of NOD2 on donor cells has no impact on GVHD pathogenicity, whereas NOD2 deficiency on only hematopoietic and not on non-hematopoietic recipient cells aggravates GVHD. *In vitro* experiments showed that the absence of NOD2 on DCs increases the expansion of alloreactive T-cells, indicating that NOD2 negatively regulates DC function and activity (22). Studies in humans proved that the relationship between this signaling pathway and GVHD is more complex. Polymorphisms in NOD2/CARD15 were identified as a risk factor in HSCT involving a HLA-identical sibling donor (177, 178), whereas in other studies of HLA-unrelated transplants, such polymorphisms adversely impacted disease relapse but not GVHD (179–181).

Overall, interactions between TLRs and NLRs have a crucial role in APC stimulation, subsequent alloreactive T-cell recruitment, and activation and aggravation of GVHD.

### IL-1 receptor signaling

The absence of TLR signaling in TLR-deficient mice does not completely abrogate GVHD (160, 182, 183). The counterpart of TLR in the TIR family is highly involved in different phases of GVHD pathogenesis. IL-1 is one of the most active pro-inflammatory cytokines in inflammatory diseases. The IL-1 receptor family contains 10 members in total. The first two members are IL-1α and IL-1β, both of which are synthesized as 31-kDa precursors (pro-IL-1α and pro-IL-1β). They are enzymatically cleaved into N-terminal prodomains (184) and are agonists for the IL-1 type 1 receptor (IL-1R1) (185). IL-1α and IL-1β seem to activate similar cellular responses upon binding IL-1R1 *in vitro*, and the main biological difference between these proteins seems to be associated with the source and presentation of the cytokine *in vivo*.

IL-1α has a central role in mediating sterile inflammation induced by cell necrosis, and it seems to be the dominant agonist in a response dependent on IL-1R1 and MyD88 signaling (186). IL-1α also has been shown to be released from dying cells (187, 188). In HSCT, IL-1α is also a dominant mediator of CD4 T-cell activation mediated by allogeneic endothelial cells expressing HLA-DR (189). IL-1β increases the expression of adhesion molecules on vascular endothelium and enhances the expression of chemokines on T-cells, thus attracting blood-borne inflammatory cells into target tissues. IL-1β also stimulates mucosal myofibroblasts and matrix metalloproteinase (MMP) release, causing tissue mucosal destruction (190–192). The IL-1 receptor antagonist (IL-1Ra) competes with the two agonist molecules for IL-1R1 binding. All three genes are located in a cluster on chromosome 2q. The fourth member of this family is IL-18, the gene for which is located on chromosome 11q (193). IL-18 was identified as a factor promoting IFN-γ production and activates TNF-α receptor-associated factor (194). It has been reported that many hematopoietic and non-hematopoietic cells produce IL-18 in inflammatory conditions (195). In HSCT, it was shown that IL-18 levels in plasma increase with acute GVHD in both human and animal models (196–199).

### IL-33 and its receptor (ST2) signaling

IL-33 (also known as IL-1F11) was identified as a new member of the IL-1 cytokine family (200). Similar to IL-1α and HMGB-1, IL-33 has dual functions, acting both as a traditional cytokine and as an intracellular NF with transcriptional regulatory properties (201). IL-33 is widely expressed in tissues, but it appears that its expression in organs is restricted. Human and murine mRNA analysis showed that IL-33 is predominantly expressed in stromal cells including fibroblasts, smooth muscle cells, epithelial cells, and endothelial cells and is largely absent in hematopoietic cells (200). IL-33 may be produced during necrosis. In apoptosis, IL-33 is cleaved by caspases-3/7, leading to inactivation of its pro-inflammatory properties. For this reason, IL-33 is considered an endogenous danger signal or alarmin (202). The only known receptor for IL-33 is ST2 (200). The ST2 gene is now known to encode at least three isoforms of ST2 proteins: the transmembrane form known as ST2L, variant ST2 (ST2V) that is mainly present in human gut (203), and secreted soluble ST2 (sST2), which serves as a decoy receptor for IL-33 to prevent IL-33 binding to and signaling through ST2L (204, 205). The strongest sST2 mRNA expression was detected in heart and lung tissues (206), the cardiovascular system, endothelial cells (207), cardiac myocytes, and fibroblasts (208). The secretory capacity of these cells for sST2 is enhanced by pro-inflammatory cytokines (TNF-α, IL-1β) or LPS (206). sST2 levels in serum were correlated with acute myocardial infarction (209) and pulmonary fibrosis (210). Recently, high levels of plasma sST2 were shown to be a risk factor of GVHD in patients after allogeneic HSCT; patients who were resistant to treatment showed elevated levels of sST2 and had higher mortality regardless of the grade of GVHD (140). This finding may allow physicians to predict disease and apply interventions earlier. It may also represent a novel therapeutic opportunity in GVHD and other related diseases.

## Organ-Specific Tissue Damage Following HSCT

### Skin

Frequently, the first presentation of GVHD involves the skin, typically manifesting initially as palmar and acral erythema, resembling a sunburn reaction, or an acute symmetric morbilliform eruption (211). The histopathology of GVHD is a lichenoid inflammatory process of the epidermis with variable numbers of lymphocytes arranged in a linear fashion along the basement membrane zone. The hallmark change is dead cells consisting of apoptotic keratinocytes, with tightly adherent lymphocytes observed in the epidermis with associated vacuolar interface changes (212). High-dose radiation activates skin DCs, which upregulates the expression of HLA-DR, adhesion molecules, co-stimulatory molecules (213), and PRRs, producing inflammatory cytokines and danger signals that contribute to skin GVHD by mediating memory T-cell recruitment to the skin (214). In cells undergoing programed cell death, activation of caspase-3 occurs as a downstream event that links both extrinsic (death receptor-mediated) and intrinsic (mitochondrial- or DNA damage-mediated) apoptotic pathways, which means that the presence of caspase-3 in the cell will not only identify it as apoptotic but also will indicate that the apoptotic machinery is involved in the premature demise of target cells. Labeling GVHD lesional skin using antibodies that recognize cleaved caspase-3 (215) identified apoptotic keratinocytes located at the base of rete ridges. Significant elevation of elafin plasma concentrations in patients with skin GVHD is due to keratinocyte damage (60).

### GI tract and liver

**Figure 4:**
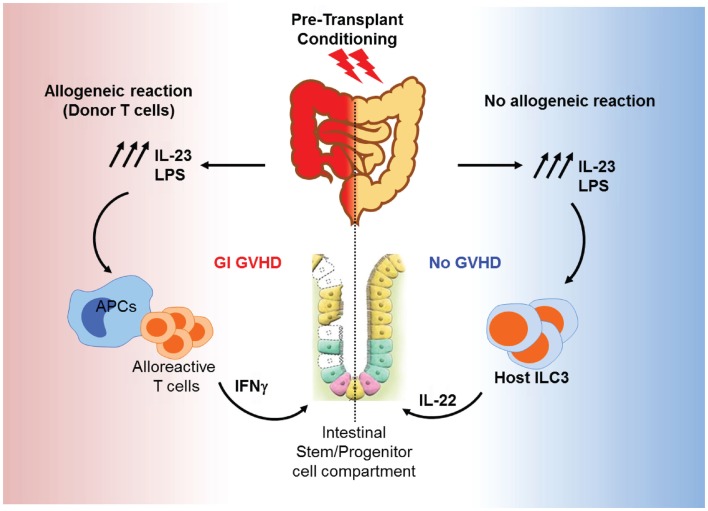
**Roles of IL-23 in GI GVHD**. Conditioning induces IL-23 production and LPS release from the GI tract. LPS and IL-23 act together to prime APCs to activate alloreactive T-cells. Activated T-cells produce IFN-γ and other inflammatory cytokines, resulting in the elimination of ILC3 and damage of the intestinal stem cells. This leads to more gut injury and GI GVHD (left). In the absence of alloreactive donor T-cells, IL-23 stimulates ILC3 to release IL-22, which protects the intestinal stem-cell compartment and promotes gut recovery from conditioning damage (right).

Gut tissue damage may be the first consequence of transplant conditioning and could be of particular significance for GVHD for two main reasons: the transplant conditioning regimen may deplete and/or alter the microbiota and epithelial barrier damage could allow for increased bacterial translocation, specifically in the gut. It is assumed that these processes lead to an increase in inflammation and exacerbate epithelial insult, as shown in an IBD model (216). Certain commensals such as *Bifidobacterium* strains may protect the host by improving the intestinal barrier. *Bifidobacterium* have carbohydrate transporters that can generate short-chain fatty acids, particularly acetate, which promotes defense functions in host epithelial cells in the distal colon (217). Recent GVHD studies have begun to analyze the dynamics of the gut flora during HSCT and how the innate immune receptors that recognize microbes may contribute to GVHD pathogenesis. In an experimental irradiation-independent non-myeloablative HSCT model, a gut microbial shift toward pro-inflammatory bacterial species was seen in mice that develop GVHD (136). It is still unclear whether the microbial changes in the gut are the cause or the result of GVHD, and whether these bacterial populations reflect endogenous microflora or overgrowth of pathogenic organisms due to the elimination of benign microbes. Endotoxin is a constituent of normal bowel flora that has the ability to stimulate the release of inflammatory cytokines that are known to be important mediators of clinical GVHD and most likely permeate the systemic circulation through the intestinal barrier, which is disrupted by the conditioning treatment (218). Also, microbial super antigens may activate B-cells by direct stimulation of MHC class II molecules (219). The early phases of changes in the GI tract have been described in animal models that do not use chemotherapy or radiation to condition the host; therefore, direct comparisons to clinical GVHD after bone marrow transplantation are not possible. The initial proliferative phase results in increased crypt cell mitotic activity, crypt lengthening, and the presence of intraepithelial lymphocytes. In experimental systems, this phase seems to be linked to IFN-γ production (220), which increases MHC class II expression and gut permeability by altering tight junction integrity and may modulate crypt stem-cell turnover (221). The histologic features of the GI tract in clinical GVHD and experimental GVHD after myeloablative conditioning are consistent with the destructive and atrophic phases, characterized by villus blunting, lamina propria inflammation, crypt destruction (with crypt stem-cell loss), and mucosal atrophy (222). Cytotoxic T lymphocytes do not appear to play a dominant role in experimental GVHD of the GI tract (215, 223–225), despite the ability of intraepithelial lymphocytes to induce Fas-mediated apoptosis of host-type tumor cells (226). It is clear, when these findings are considered in aggregate, that cytokines and cellular effectors combine to produce the specific damage to target organs as well as the systemic toxicity of acute GVHD. Furthermore, the absence of GVHD toxicity in other visceral organs, such as the kidney (currently debated), argues against circulating cytokines as the sole cause of tissue-specific damage. The infiltrates seen in GVHD target organs are generally thought to consist of T-cells responding to alloantigens presented by host tissues. LPS leakage through the skin or mucosa may act as an adjuvant to the antigens expressed in these tissues, attracting and activating alloreactive donor T-cells. In BMT models, LPS levels increase progressively during the first 4 weeks post-BMT. These levels lead to aggravated disease severity through TLR4 signaling, which induces inflammatory cytokine production. Deficiency of TLR4 on donor bone marrow cells reduces colonic GVHD severity. Interestingly, this reduction in GVHD severity was accompanied with a decrease of IL-23 levels. On the other hand, mice receiving allogeneic bone marrow from IL-23 knockout mice demonstrated less colonic pathology, and low levels of colonic LPS compared to wild-type controls. Interestingly, IL-17 was not detectable in the colon, while IFN-γ was markedly increased, in association with the LPS/IL-23 feedback loop (227). In consequence, IFN-γ activated macrophages after exposure to LPS release a significant amounts of inflammatory cytokines in GI tract but not in other target organs of GVHD (218). IL-23 could also enhance host IL-22-producing type 3 innate lymphoid cells (ILC3s) and promote gut recovery after conditioning. Alloreaction generated by donor T-cells damages the gut stem-cell compartment and eliminates recipient IL-22 producing ILC3, which are the main source of IL-22 in the gut and are known to protect intestinal stem cells (228). A deficiency in ILC3s leads to severe liver and GI GVHD and increases mortality. Moreover, a recent clinical study showed clear correlation between activation and expansion of intestinal ILC3s and the absence of acute GVHD (229). Figure 4 shows a hypothetical model for the roles of IL-23 in GI GVHD. Thus, reductions in the doses of chemoradiotherapy to condition bone marrow transplant recipients have reduced the incidence of GVHD, as demonstrated in experimental models (230, 231). This reduction is the result of reduced priming of mononuclear cells by lower doses of total body irradiation (TBI) and subsequent reductions in TNF-α production (224). Intestinal and liver tissue damage leads to the release of soluble mediators that correlate positively with GI and liver GVHD pathology. Significant augmentation of liver epithelial marker cytokeratin-18 protein (CK18) (232) has been demonstrated in serum from patients with hepatic and GI GVHD. Interestingly, CK18 levels begin to increase before the clinical manifestation of GVHD in some patients. Also, CK18 levels were correlated with bilirubin (liver function marker) levels. This correlation is specific to hepato-intestinal GVHD (233). RegIIIα levels were also significantly increased in patients with GI GVHD as mentioned above (105, 234). It is well known that Reg proteins act downstream of IL-22, which protects the function of intestinal mucosa, intestinal stem cells, and ILC3s (235, 236).

### Thymus

Donor CD8 T-cells can damage medullary epithelial cells in the thymus and cause the generation of donor alloreactive CD4 T-cells (237), which suggests that GVHD autoimmunity can appear during acute GVHD after allogeneic HSCT. The thymic epithelial damage caused by anti-host reactive T-cells impairs negative selection in the thymus, consequently leading to the presence of autoreactive T-cells in the periphery (211, 238, 239). These autoreactive T-cells are specific and able to recognize both self (donor) and host MHC class II antigens. Thus, they may participate in acute allogeneic GVHD and are thought to underlie the pathophysiology of chronic GVHD with its clinical autoimmune manifestations (214, 238). Animal studies revealed that the presence of the thymus is required for the development of autologous GVHD, based on comparisons with thymectomized rats before transplantation (240). The thymus is a primary target organ of GVHD. Thymus toxicity is generated further by regimen preparation (radiation) and age-associated thymus toxicities, resulting in thymus dysfunction and GVHD-induced damage of lymphopoietic microenvironments. However, keratinocyte growth factor (KGF) has promising effects for preventing GVHD-mediated thymic damage (241). Reduced thymic function is detrimental for thymopoiesis recovery, and insufficient recovery of thymopoiesis is directly linked to opportunistic infections and adverse clinical outcomes in recipients (242). Thymic GVHD damages the architecture and composition of the thymic environment (243). In the thymus stromal and epithelial cells, growth factors are produced locally including IL-7 and stem-cell factor (SCF), as are chemokines involved in T-cell precursor migration such as CCL9 and CCL21. Thymic GVHD affects T-cell renewal and differentiation, and in humans, it has been reported that when thymic function is transiently impaired in young patients because of acute GVHD, there are decreases in TCR-β chain rearrangement circles (244, 245).

### Oral

The overall incidence of oral complications after bone marrow transplantation is 80%. Most oral complications are resolved in autologous HSCT within 6 months post-transplantation. Oral complications in allogeneic transplantation include mucositis, oral dryness, taste change, and infection, and all symptoms are associated with GVHD (246–248). These oral changes are painful and impair patients’ quality of life (249, 250). The initiation of oral mucositis is induced by DNA and non-DNA damage caused by ROS generated by damaged basal epithelial cells, endothelial cells, and submucosal cells, in particular (251). This leads to NF-κB activation, inducing adhesion molecule expression, and MAPK and COX2 activation, resulting in the generation of IL-1β, IL-6, and TNF-α (252–254). This reinforces NF-κB activation and amplifies the response via the over-production of inflammatory cytokines (255–259). Perturbation in the immune response to the microbiota leads to spontaneous inflammation, and vice versa, changes in microbiota diversity are associated with pro-inflammatory states (260, 261). In HSCT recipients, substitution with coagulase-negative *Staphylococci* for *Streptococci* is associated with oral mucositis (137). It was suggested that subcutaneous administration of IL-11 reduces the severity of oral mucositis by maintaining keratin production in epithelial cells and reducing mucosal pro-inflammatory cytokine expression (262). However, it causes severe fluid retention and early mortality in clinical trials (263). Administration of TGF-β3 prior to chemotherapy down-regulates epithelial cell expansion and reduces oral mucositis in hamsters (264). KGF promotes upregulation of Bcl2 and cell survival (265) as well as upregulates IL-13 that attenuates TNF-α (266). KGF has beneficial effects on oral mucositis prevention in high-dose chemotherapy and TBI-treated patients (259, 267).

## Therapeutic Approaches to Target Danger Signals

Since the late 1980s, different therapeutic approaches have been established to overcome GVHD (268, 269). These classical therapeutic strategies target only donor T-cells by inhibition or even depletion and may impact the immune reconstitution and graft-versus-leukemia effect. Recent therapeutic strategies have focused on PAMPs and/or DAMPs released by the host after tissue damage, which are the first triggers of activation of host APCs and donor T-cells. Therefore, targeting PAMPs and DAMPs may not impair donor cell function, immune reconstitution, or anti-tumoral activity. Here, we highlight therapeutic strategies targeting danger signal or alarmins.

### Siglec-G ligand

Recently, it has been shown that conditioning inhibits the expression of Siglec-G, which could be activated by HMGB-1. The absence of Siglec-G ligand (CD24) increases susceptibility to GVHD in mice because of the response to DAMPs, but not PAMPs. Administration of CD24 fusion protein led to a reduction in GVHD severity and mortality. This approach reveals the importance of targeting DAMPs separately from exogenous PAMPs in GVHD therapy (39).

### Alpha-1 antitrypsin

Heparan sulfate, as described above, is detected in patient sera following HSCT. Murine studies showed that heparan sulfate levels are reduced significantly upon the use of the elastase inhibitor alpha-1 antitrypsin (270), and this reduction in heparan sulfate was correlated with reduced GVHD severity in murine models. The alteration of heparan sulfate in mice treated with alpha-1 antitrypsin subsequently led to a reduction in inflammatory cytokines such as TNF-α and IL-1β and enhanced IL-10 production and Treg expansion. Another study showed that alpha-1 antitrypsin treatment suppresses IL-32 expression in T-cells (271). Clinical trials using alpha-1 antitrypsin as GVHD prophylaxis are currently underway.

### ILC3s

Many recent studies have emphasized the importance of RORγt ILCs in regulating mucosal immune responses via the control of intestinal microflora expansion and composition and regulation of CD4 T-cells (272). Depletion of Nfil3 in mice dramatically impairs the number of IL-22-producing ILC3s, which resulted in compromised innate intestinal immune defense against bacterial infection (273). IL-22-producing ILC3s have a beneficial impact on gut protection in metabolic disorders, improving insulin sensitivity as well as preserving the gut mucosal barrier and endocrine function (274). As mentioned before, IL-22-producing ILC3s have a crucial role in reducing epithelial and intestinal stem-cell damage and reducing GVHD severity and mortality (228). The same research group has demonstrated that daily administration of IL-22 for 3 weeks starting 1 week post-transplantation increases the survival and function of host radio-resistant ILC3s, subsequently reducing apoptosis in host intestinal stem cells and reducing GVHD severity through preservation of host cells from damage without compromising immune function or reconstitution (275). This makes IL-22 administration one of most promising therapies for GI GVHD. The transcription factor aryl hydrocarbon receptor (AhR) is highly expressed in ILC3s and is required for ILC3 development (276), particularly IL-22-producing ILC3s (277). Thus, using small molecules that activate AhR might be a promising future therapeutic strategy in preventing GI GVHD and ameliorating immune functions post-transplantation as well.

### IL-1 receptor antagonist

Blockade of IL-1 with IL-1 receptor antagonist significantly reduces mortality from experimental GVHD and enhances engraftment of HSCs (278). In a clinical trial, IL-1 receptor antagonist reduced the severity of GVHD (279), but when used as prophylaxis, no significant impact on GVHD was observed (280).

### Anti-ST2 or IL-33

A high level of sST2 is a risk factor for GVHD (140), with levels of sST2 positively correlating with high rates of mortality in patients with GVHD. Other studies reported that sST2 is associated with cardiovascular mortality. sST2 concentrations have been linked with inflammatory markers (281), and sST2 has been associated with disease severity in pulmonary arterial hypertension (282). sST2 is rapidly synthesized and released by endothelial cells in inflammatory conditions and in the setting of tissue damage (207). sST2 acts as a decoy receptor for IL-33, inhibiting signaling through membrane ST2 (ST2L) (283). In murine models, exposure of murine T-cells to sST2 inhibits TH2 cytokine production and shifts the cells toward a TH1 response (283). It is possible that high levels of sST2 after tissue damage induced by conditioning leads to activation of donor T-cells toward a type 1 response, thereby increasing GVHD responses. It is also possible that inhibition of sST2 decreases this phenomenon and thus increases type 2 T-cell responses. Another possible therapeutic approach would be to increase the expression of ST2L and make it more available for IL-33 binding and signaling. Both strategies should not impair the immune response of donor cells, but will work by limiting the effects of host tissue damage, opening new robust therapy options for GVHD.

### Mesenchymal stem cells

Mesenchymal stem cells (MSCs) are multipotent mesenchymal stromal cells with fibroblastic-like morphology that can differentiate into bone, cartilage, or fat cells (284). These cells have the capacity for non-specific immunosuppression and immunomodulation. It has been shown that infusion of MSCs in high-risk major-mismatched transplant recipients reduces the incidence of life-threatening GVHD (285, 286). Recent clinical studies showed that weekly infusion of MSCs with a fixed dose for 3 weeks reduced significantly the severity of the disease; specifically, patients with steroid-refectory acute GVHD experienced a complete response. Clinical response was correlated with a significant decline in RegIIIα and elafin GVHD biomarkers (287).

### Anti-TLRs

The expression of PRRs at the epithelial surfaces is equally important as that in immune cells in combating or facilitating entry of organisms into the body, including bacterial translocation from the gut after irradiation (288). Activation of PRRs results in additional vascular damage and infiltration of inflammatory cells that creates a cascade of lesions in a pro-oxidant microenvironment, aggravating tissue damage and causing a “danger” zone (289). An antagonist of LPS, the TLR4 ligand, results in reduced intestinal damage and GVHD severity without altering donor T-cell activity to the host antigen (133). Novel anti-TLR antibodies particularly anti-TLR4 and anti-TLR2 are being developed (290) and will soon represent a novel class of potential therapeutics for GVHD treatment.

## Chronic GVHD and Danger Signals

**Figure 5:**
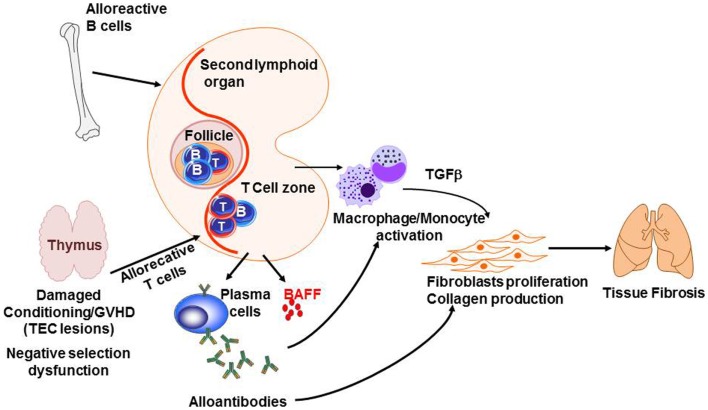
**Pathogenesis of chronic GVHD**. The thymic epithelial cells (TECs) are damaged by alloreactive T-cells leading to impaired negative selection. In addition, alloreactive B- and T-cells cross talk leading to sBAFF release and production of alloantibodies by plasma cells. At the same time, cytokines and chemokines produced by B- and T-cells activate macrophages and monocytes. Together, antibodies and TGFβ induce fibroblasts proliferation and activation as well as collagen production, which results in fibrosis in target organs such as the lungs.

Graft-versus-host disease studies have led to a decrease of early mortality in related-allogeneic HSCTs, but late long-term morbidity and mortality caused by chronic GVHD remains a major challenge (291). The pathogenesis of chronic GVHD is complex and poorly understood, but is likely to involve dysfunction of tolerance determining mechanisms similar to classic autoimmune diseases. Figure 5 summarizes some of the knowledge of the pathophysiology of chronic GVHD. Briefly, negative selection in the thymus is impaired because of thymic epithelial cell (TEC) damage after allogeneic reaction. In addition, the cross talk between alloreactive T-cells and B-cells enhances B-cell activating factor (BAFF) release and production of alloantibodies, which, together with cytokines and chemokines produced by T-cells and B-cells, activates macrophages and induces proliferation and activation of fibroblast and collagen production, resulting in tissue fibrosis. Very few studies showed a direct impact of PAMPs or DAMPs in chronic GVHD. It has been shown that LPS enhances peribronchiolar fibrosis in synergy with TH17 production and leads to chronic pulmonary GVHD (292). However, a clear indirect effect is the altered T- and B-cell homeostasis. Patients with chronic GVHD showed inverted ratio of CD4:CD8 (293). CD4+ regulatory T-cell frequency was dramatically decreased in these patients comparing to patients without active chronic GVHD. This reduction in Tregs:Tcon ratio was explained as following: (i) Tregs acquire a predominately effector memory phenotype (294), (ii) under lymphopenia-induced expansion, CD4+ Tregs proliferate more than conventional T-cells, which increases Treg susceptibility to Fas-mediated apoptosis (295), and (iii) progressive loss of Aire expression by TEC, which is crucial for naive Treg generation (294). In chronic GVHD, critical breakdown in peripheral B-cell tolerance was shown. Among patients with cGVHD, BAFF reaches a persistently high level (296). Chronic exposure to BAFF results in elevated basal expression of the proximal signaling components B-cell linker protein (BLNK) and Syk, which may contribute to increased responsiveness of BCR stimulation (297). Murine models of cGVHD also provided insights showing that unrestrained T follicular helper cells and germinal center B-cells are abnormally increased and strongly correlate with the development of cGVHD (298). Together, chronic GVHD is likely caused by a lack of central tolerance involving thymus dysfunction, disequilibrium of T-reg/Tcon balance, and alloantibodies generated by alloreactive B-cells.

## In a Nutshell: PAMPs and DAMPs in the Pathogenesis of Acute GVHD

Allogeneic HSCT conditioning elicits the first signal of tissue damage by releasing PAMPs, such as intestinal microflora and LPS, and DAMPs, such as S100 proteins, uric acid, HSP, and ATP. These PAMPs and DAMPs are then detected by host innate immune cells, including non-hematopoietic APCs, through PRRs (TLRs and NLRs) leading to downstream signaling through NF-κB, upregulating co-stimulatory molecules, and producing the inflammatory cytokines TNF-α, IL-12, and IL-6. Stimulation of allogeneic donor T-cells by activated host APCs in the proinflammatory environment leads to T-cells expansion and polarization toward TH1, TC1, TH17, and TC17, the key mediators of GVHD pathogenesis. These T-cells produce more inflammatory cytokines, such as IFN-γ, TNF-α, and IL-17, leading to increased tissue damage. As a consequence of damage exacerbation, more DAMPs are released from damaged tissue, such as elafin in skin GVHD, Reg3α in GI GVHD, or soluble ST2, and again newly amplify type 1 response of T-cells creating more severe pathogenicity as shown in Figure 1. Importantly, these molecules can serve as biomarkers for GVHD diagnosis and severity. These molecules may also represent a novel class of therapeutics for GVHD with the possible advantage of not altering the immune reconstitution and T-cell responses against tumors.

## Conflict of Interest Statement

The authors declare that the research was conducted in the absence of any commercial or financial relationships that could be construed as a potential conflict of interest.
